# Cranioplasty with three-dimensional customised mould for polymethylmethacrylate implant: a series of 16 consecutive patients with cost-effectiveness consideration

**DOI:** 10.1186/s41205-021-00096-7

**Published:** 2021-02-06

**Authors:** Erasmo Barros da Silva Júnior, Afonso Henrique de Aragão, Marcelo de Paula Loureiro, Caetano Silva Lobo, Ana Flávia Oliveti, Rafael Martinelli de Oliveira, Ricardo Ramina

**Affiliations:** 1 Department of Neurosurgery, Instituto de Neurologia de Curitiba, Jeremias Maciel Perretto, 300 - Campo Comprido, Curitiba, Paraná 81210-310 Brazil; 2 Post-Graduation Department of Industrial Biotechnology, Universidade Positivo, Curitiba, Brazil; 3 BMR Medical, Curitiba, Brazil

**Keywords:** 3D printing, Cranioplasty, Customised implant, Polymethylmethacrylate, Reconstructive surgery, Three-dimensional template

## Abstract

**Background:**

Different methods of cranioplasty for the reconstruction of bony skull defects exist. In the absence of the autologous bone flap, a customised manufactured implant may be the optimal choice, but this implant has several limitations regarding its technical standardisation and better cost-effectiveness.

**Methods:**

This study presents a series of 16 consecutive patients who had undergone cranioplasty with customised three-dimensional (3D) template moulds for polymethylmethacrylate (PMMA) implants manufactured after 3D modelling on a specific workstation. The virtual images were transformed into a two-piece physical model using a 3D printer for the biomaterials. PMMA implant was produced intraoperatively with the custom mould. Cosmetic results were analysed by comparing pre- and postoperative 3D computed tomography (CT) images and asking if the patient was satisfied with the result.

**Results:**

The average total time for planning and production of customised mould was 10 days. The 16 patients were satisfied with the result, and CT images presented harmonious symmetry when comparing pre- and postoperative scans. Cases of postoperative infection, bleeding, or reoperation in this series were not observed.

**Conclusion:**

Cranioplasty with high-technology customised 3D moulds for PMMA implants can allow for an aesthetic reconstruction with a fast and cost-effective manufacturing process and possibly with low complication rates.

## Introduction

Cranioplasty is a reconstructive surgery that has attracted the attention of doctors and researchers for a long time and is still one of the most commonly performed neurosurgical procedures worldwide. For over 5000 years, surgeons have been trying to determine a suitable material for the proper repair of cranial defects. A notable example is the cranioplasty of a Peruvian skull from 2000 BC; the skull was found to have a left frontal defect covered with a 1-mm-thick gold plate. At that time, the material used for the repair directly reflected the patient’s social level. This incessant search for a perfect material that provides a good functional and aesthetic result is observed even today [1].

Extensive cranial defects can occur owing to traumatic injuries, infections, congenital or neoplastic diseases, and decompressive craniectomy (DC). Cranioplasty restores the cosmetic form of the cranium to avoid post-craniectomy complications such as seizures, syndrome of the trephined, and brain herniation through the defect [2, 3].

Several techniques are available for cranioplasty. The first is the use of autogenous bone flap removed from the patient and kept in the subcutaneous abdominal pocket or preserved using the deep freezer, but the risks of infection, absorption, and reduced strength in these cases should be considered [4, 5]. The utilisation of bone grafts from cadavers (allograft) or other types of animals (xenograft) has high complication rates and is considered obsolete [1].

Alloplastic reconstruction utilising biocompatible materials has been proven to be a reliable method when an autologous bone is not available. This material should be resistant to infection, inert, noncarcinogenic, malleable, strong, easily handled, and cost-effective [6]. Different biomaterials are used for cranioplasties, often based on the routine of the institution or on the personal experience of the surgeon. Polymethylmethacrylate (PMMA), hydroxyapatite, titanium, bioactive glass ceramics, and polyetheretherketone are the most available options, each with their respective advantages and disadvantages [1, 6–8].

PMMA was first introduced after World War II, and it is biocompatible, malleable, and heat resistant with good strength; also known as acrylic, it has been widely used in cranioplasties for decades. Implant moulding occurs intraoperatively and is performed freehand by the neurosurgeon; it requires significant clinical skill and three-dimensional (3D) orientation to obtain a reasonable aesthetic result [9, 10].

In the last two decades [8, 11–13], custom implant production based on 3D computed tomography (CT) with computer-aided design/computer-aided manufacturing (CAD/CAM) has been constantly refined, aiming for a precise and aesthetic fit over the cranial defect. The ideal implant biomaterial continues to be extensively researched, and several options have been used with similar results [14–17]. The current article describes the confection of a high-technology two-piece mould with acceptable costs customised according to the patient’s bone defect to perform intraoperative PMMA modelling.

## Methods

This observational non-experimental cohort study was conducted as a retrospective analysis of prospective collected data in four parts: the Neurosurgery Department of the *Instituto de Neurologia de Curitiba*, a Brazilian health technology start-up, a postgraduate team in Biotechnology at the *Universidade Positivo*, and a Brazilian International Organization of Standardization 13,485 certified surgical products company.

Sixteen (7, female; 9, male) patients with large cranial defects were selected at the *Instituto de Neurologia de Curitiba* between May 2018 and September 2020. Indications for customised cranioplasty were DC in thirteen patients, infection of a polyetheretherketone implant in one patient, and tumour infiltration in two patients. All surgeries were performed a minimum of 2 months after the initial craniotomy. This study complied with ethical standards, and patients or members of their families provided informed consent for inclusion in the study.

### Preoperative care

Every patient underwent a high-resolution CT scan (1.25-mm slice thickness). Bone kernel reconstruction was selected to facilitate the removal of CT artifacts, such as bone spicules, and to view details of the craniectomy borders and cranial sutures. These images were shared through cloud from the Radiology Department at the *Instituto de Neurologia de Curitiba* to the engineering/design team.

### CAD/CAM treatment

**Fig. 1:**
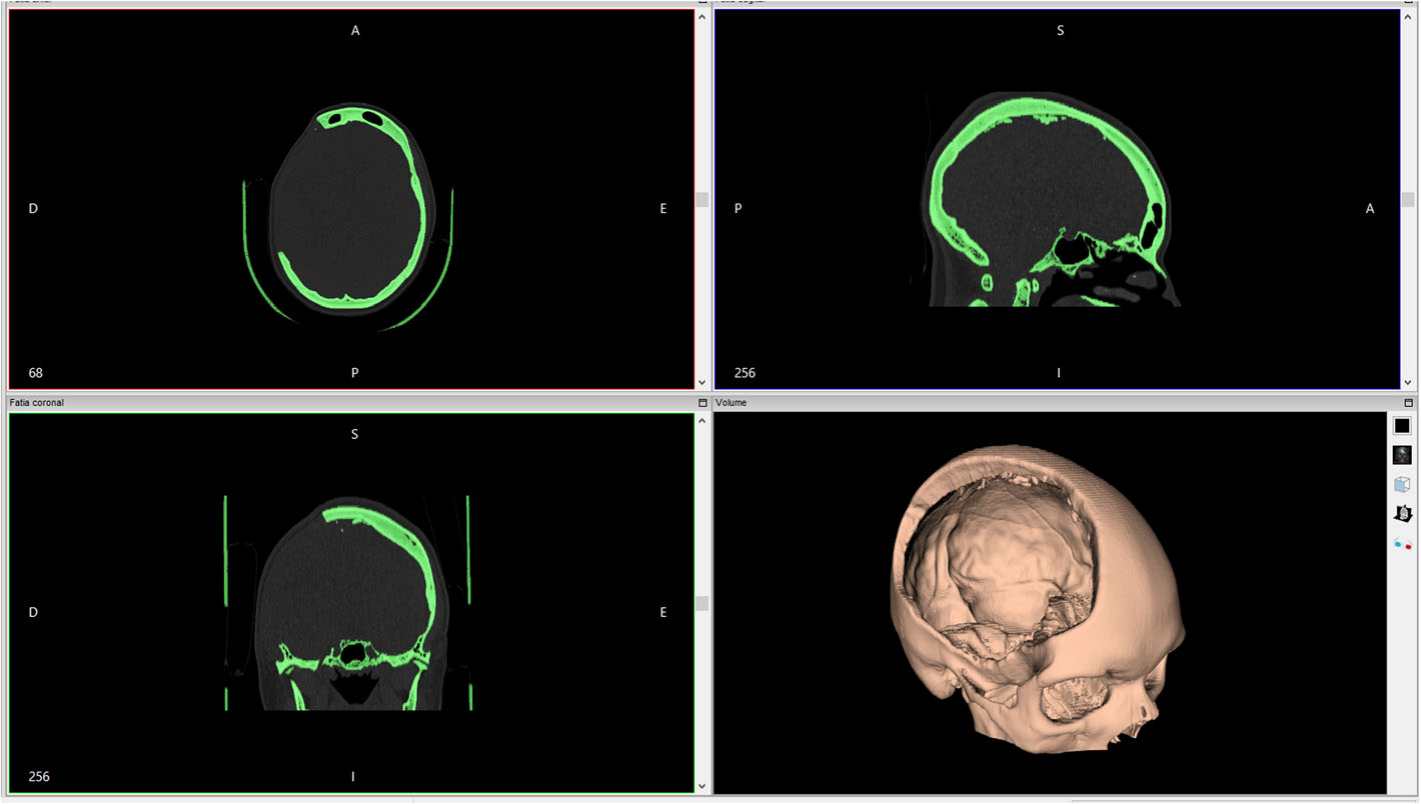
Three-dimensional rendering with computed tomography scan

CT images were exported in Digital Imaging and Communications in Medicine (DICOM) files. Subsequently, 3D reconstruction software was used to render the images (Fig. 1), reproducing the patient’s bone skull three-dimensionally. The software used for converting DICOM files to stereolithography (STL) file images was *InVesalius* 3.1 open-source software for the reconstruction of CT and magnetic resonance images (https://invesalius.github.io/), free of charge, developed by Brazilian Paulo Amorim in partnership with the Renato Archer Information Technology Center.

**Fig. 2:**
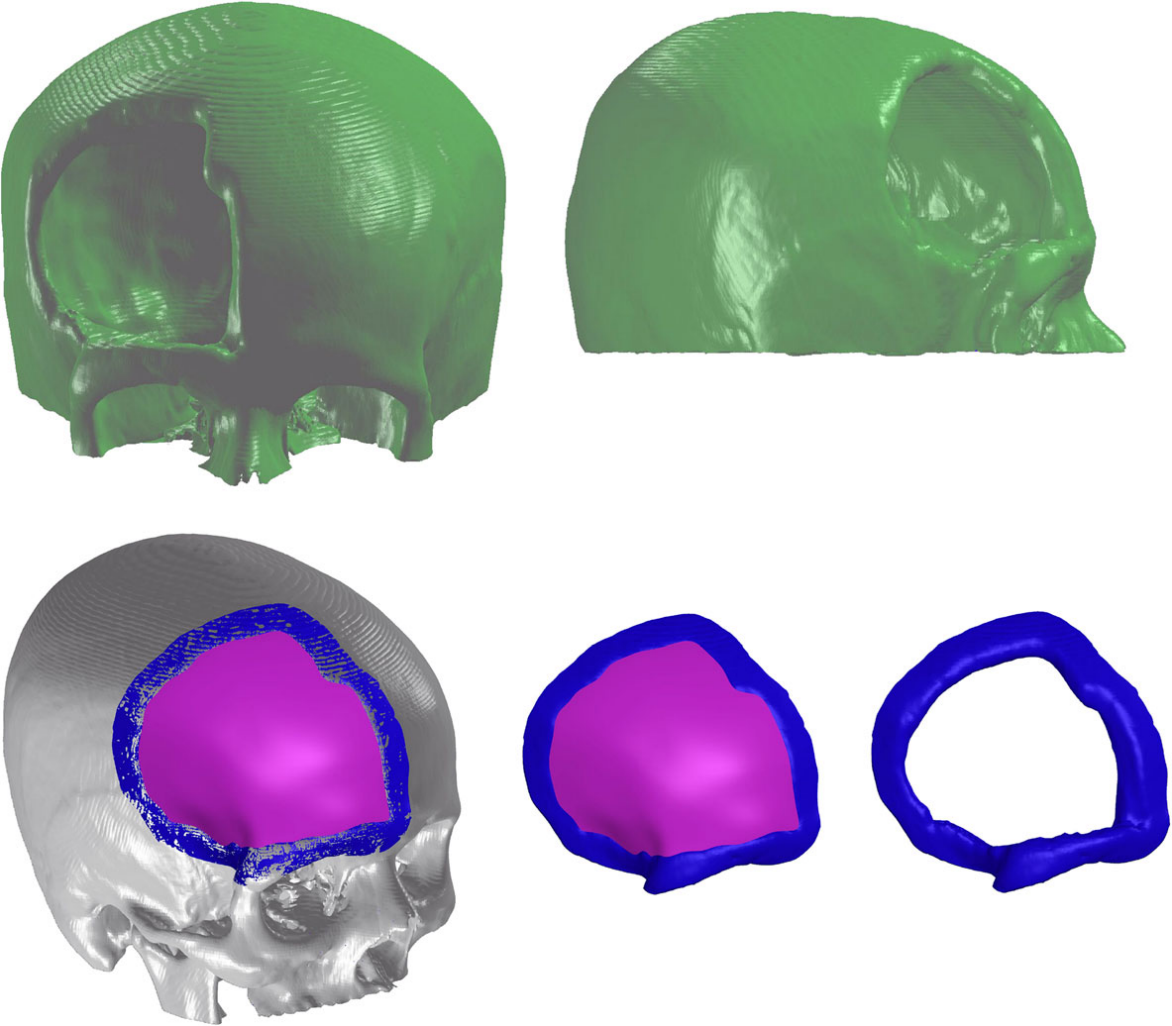
3D model reconstruction in coronal (upper left) and sagittal (upper right) views. Lower images show reconstructed skull with craniectomy margins (blue) and implant sketch (pink)

With generated rendering, a 3D parametric modelling software (SOLIDWORKS 2020 SP2.0, Dassault Systèmes) was used to treat the cranial defect. This software is parametric and classified as a “middle engineer” and has several tools for modelling 3D geometries. Skull defects were isolated and edited with surface treatment tools for detailing and refinement aesthetically and geometrically compatible with the real skull defect (Fig. 2). New bone contours were created by two methods: 1. mirroring of the normal side, in cases of unilateral defect; or 2. using patient CT images prior craniectomy, when available.

**Fig. 3:**
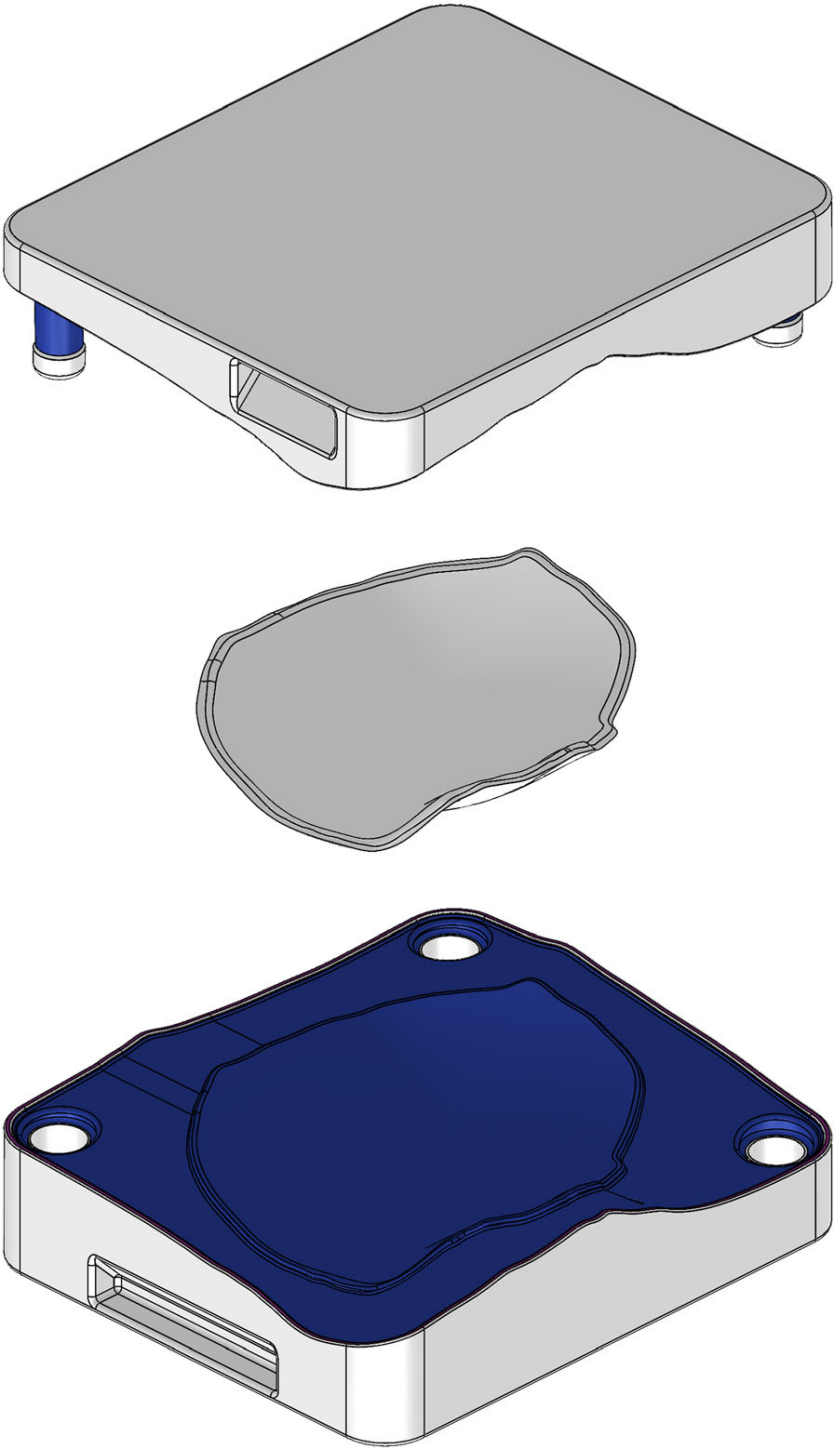
Two-piece mould template with a modelled implant in the middle

The prosthesis was modelled based on an isolated defect. The defect was geometrically filled through the modelling tools, generating a 3D model of the intended prosthesis (Fig. 3). Planning approval by the neurosurgical team was required before triggering the CAM process.

**Fig. 4:**
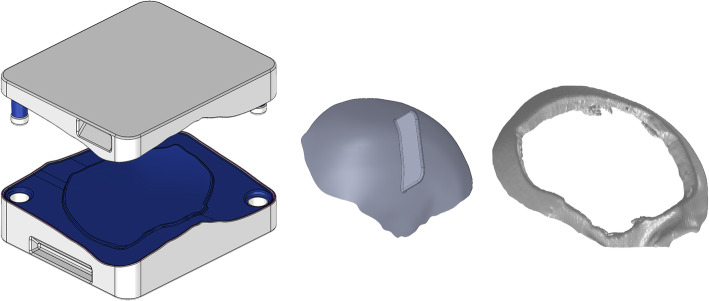
Left to right: two-piece mould template, test implant and printed defect

A custom resection template was made for patients 9 and 11, both of them cases of intraosseous lesions, delimiting the area for a single-step frame-guided resection and cranial reconstruction. Pre-operative planning required both neurosurgeon and engineer to determine the exact limits of the bone removal area (Fig. 4). This additional frame template was positioned and temporarily fixed with three small screws over the skull, based on the identification of the relevant cranial sutures.

### Mould and test implant printing

**Fig. 5:**
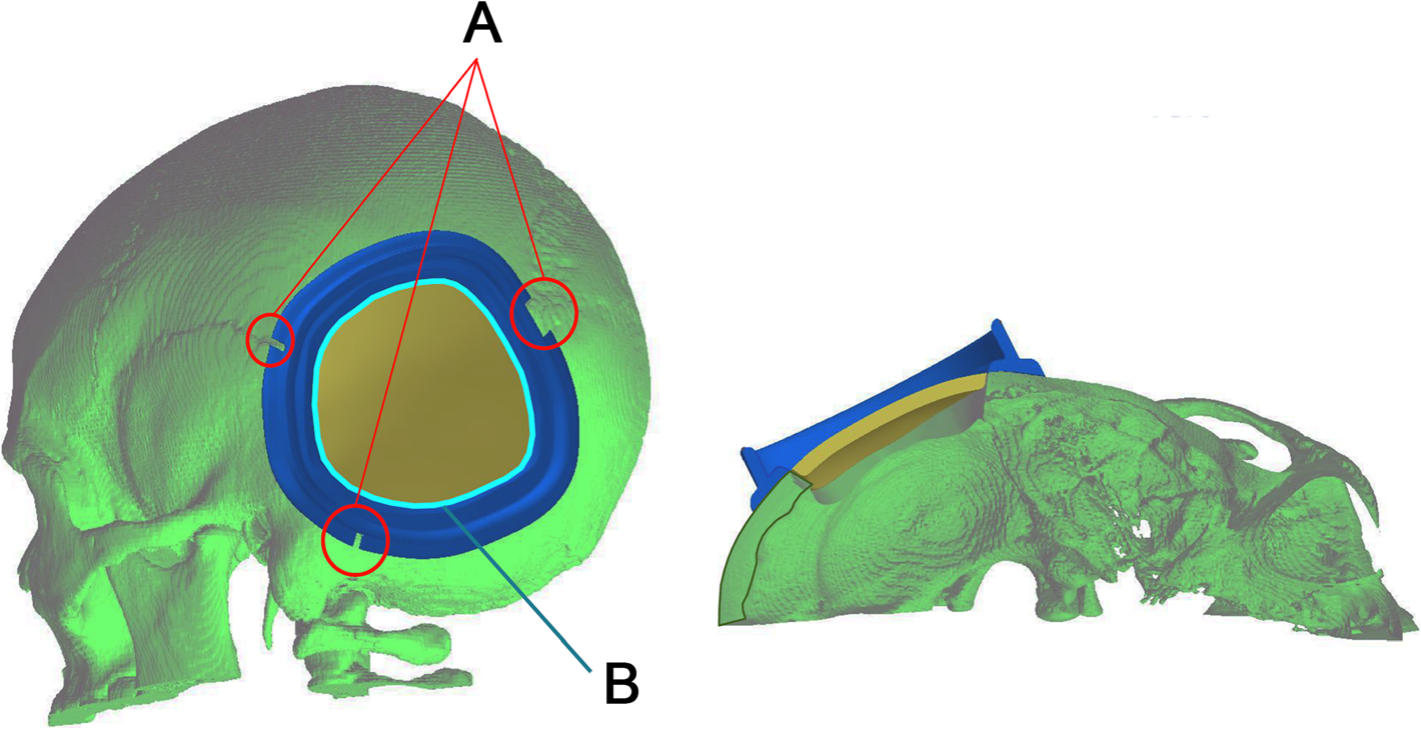
Template frame for single-step frame-guided resection: A. placement guided by cranial sutures; B. bone removal area

After completion and approval of virtual treatment, the STL extension file was sent to a 3D printer slicing application to enable production. A craniectomy defect model, test implant, and two-piece mould templates were printed with Stratasys, Ltd. fused deposition modelling technology (Fig. 5). All templates were printed in polycarbonate. A medical grade silicone layer was applied over the modelling surface of the two-piece mould to prevent the implant from sticking. Each printed piece was properly identified. After manufacture, the templates were cleaned and sent to the hospital. Full production, from CAD/CAM to delivery, took 10–13 days.

### Intraoperative management

**Fig. 6:**
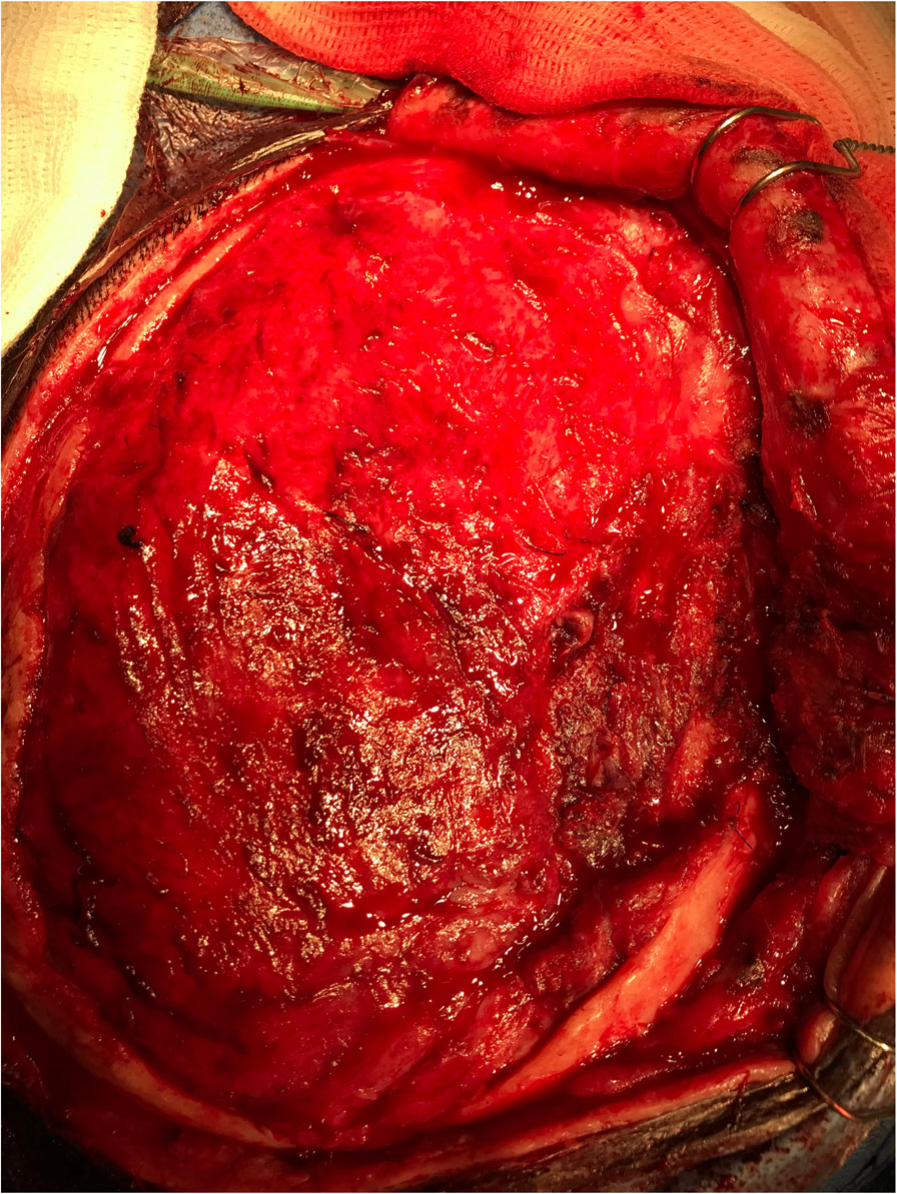
Craniectomy borders

**Fig. 7:**
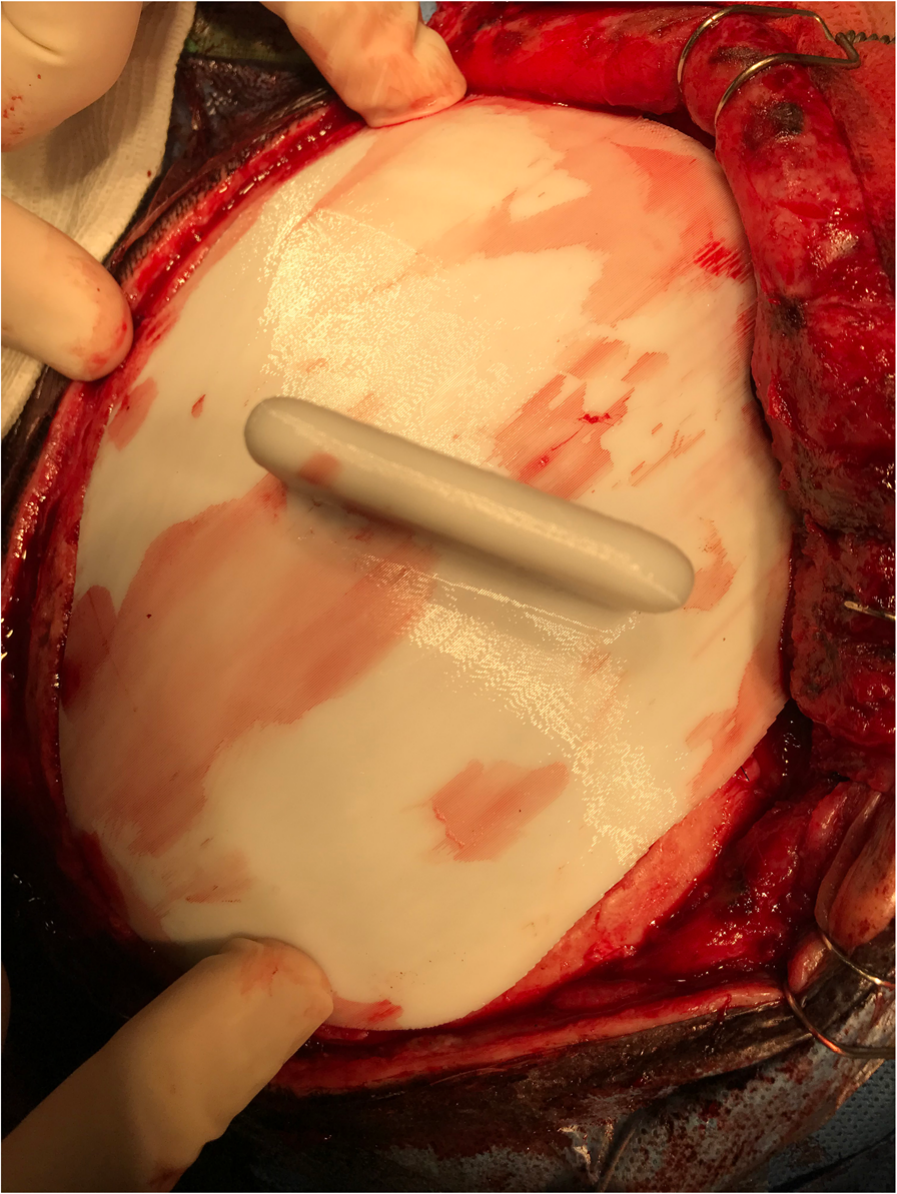
Test implant over a defect

**Fig. 8:**
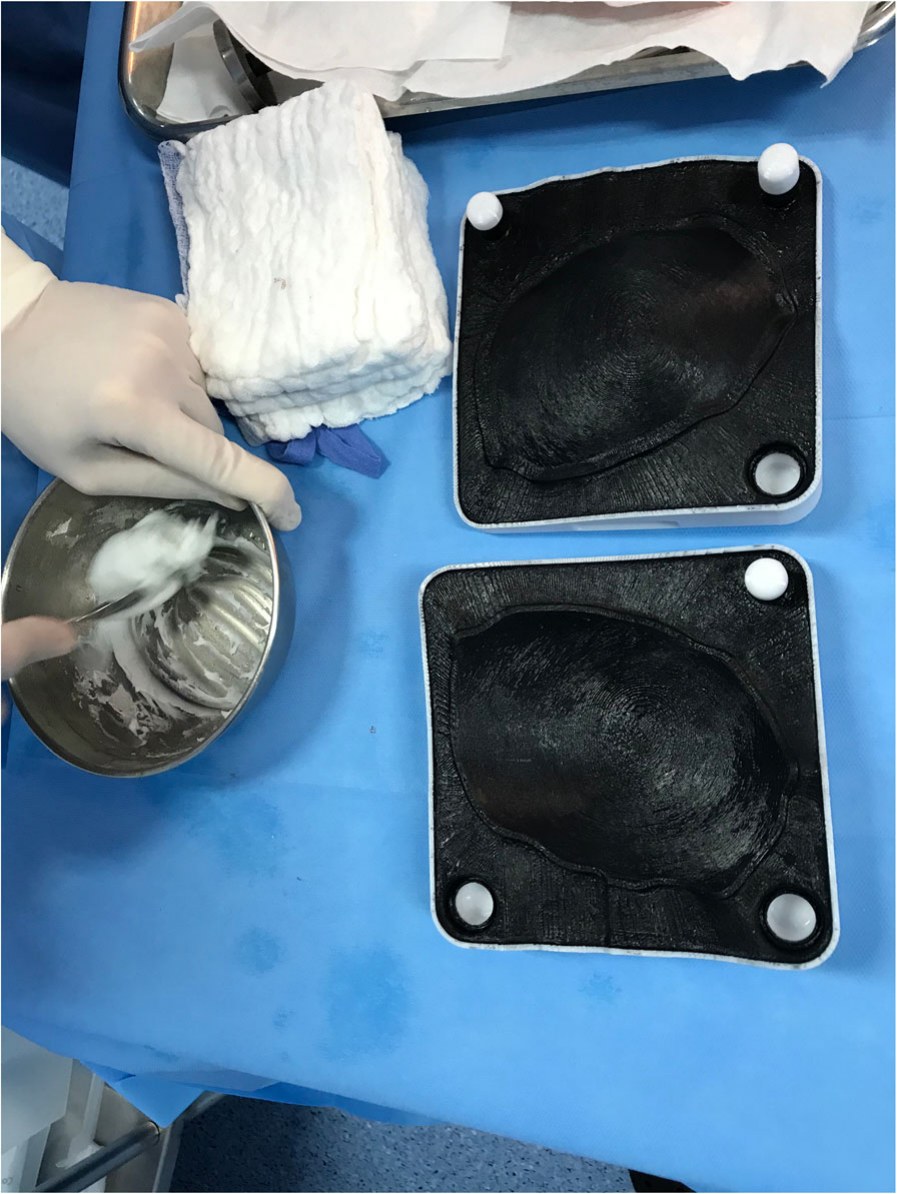
Polymethylmethacrylate preparation in a two-piece mould

**Fig. 9:**
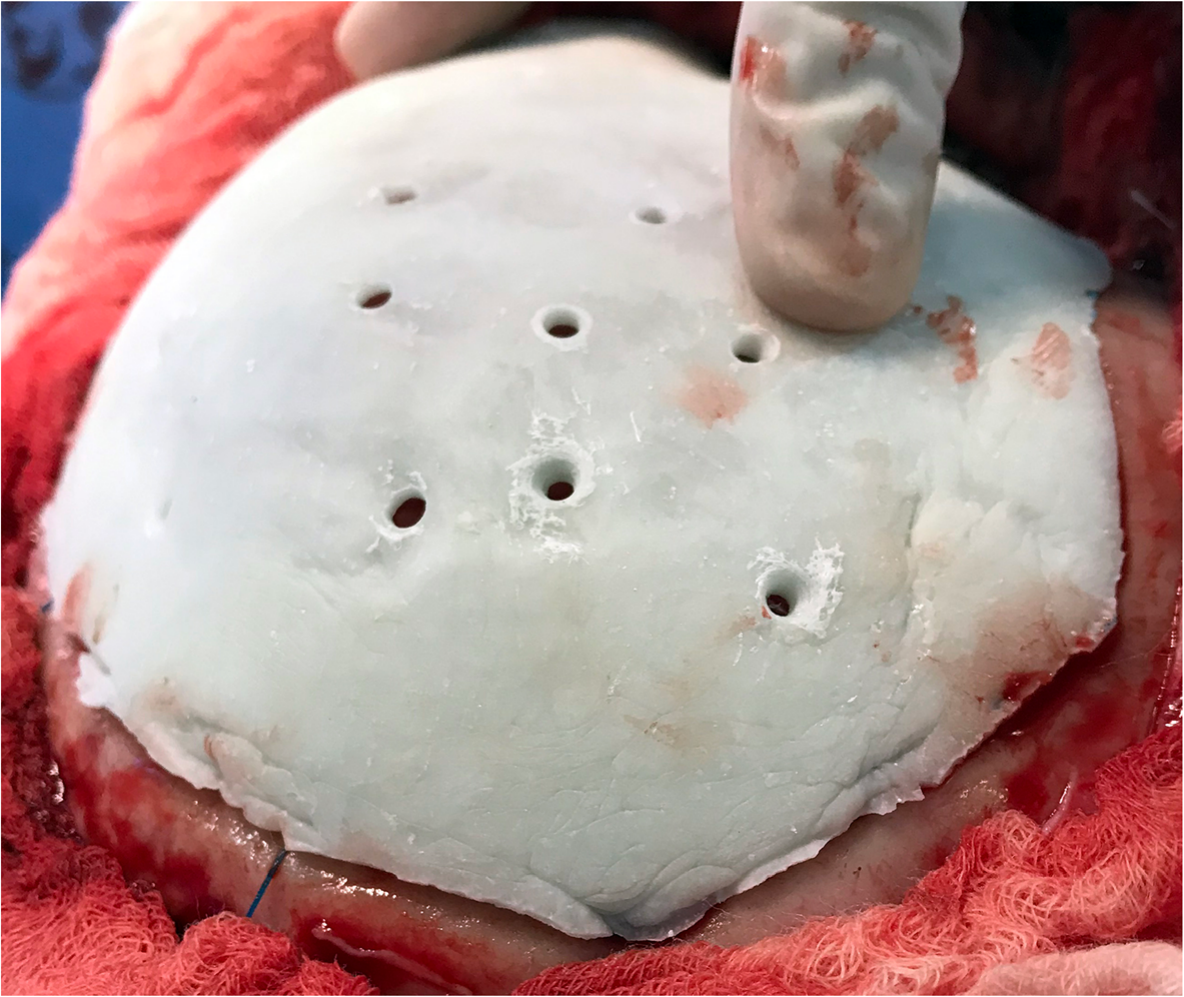
Implant placed over a defect with small holes

**Fig. 10:**
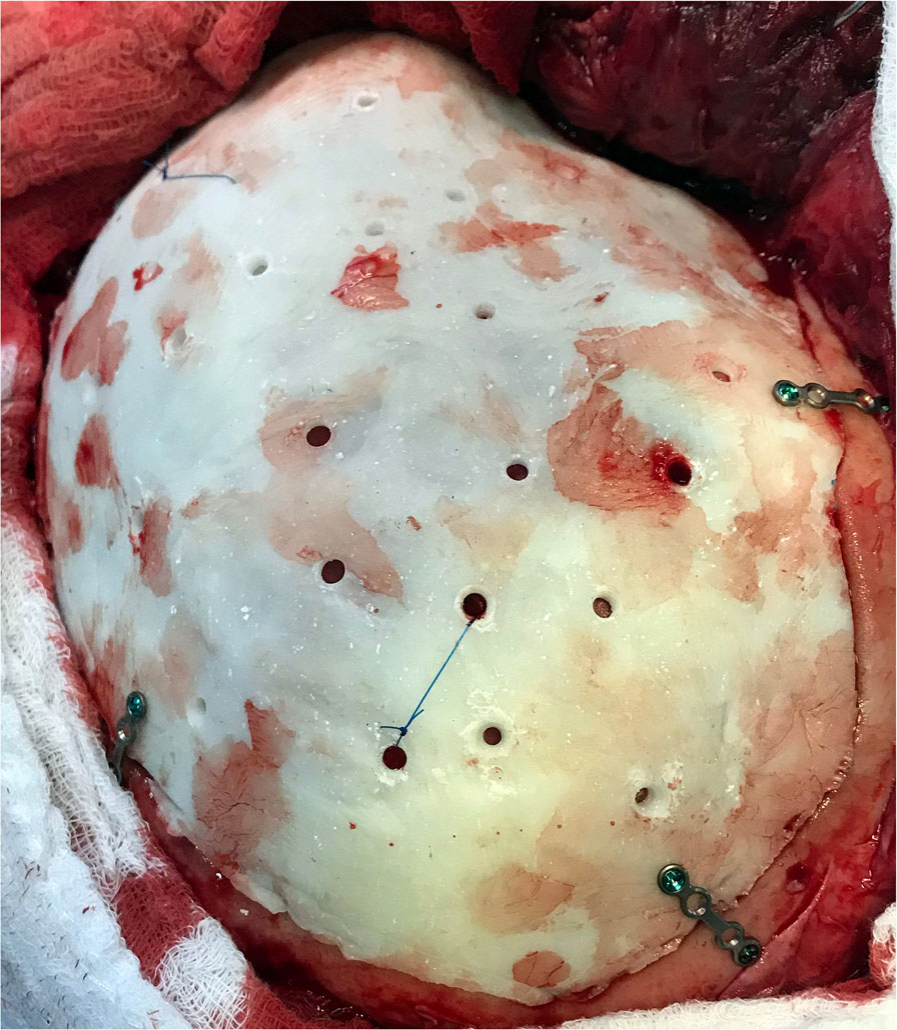
Implant fixed over a defect with mini titanium plates

**Fig. 11:**
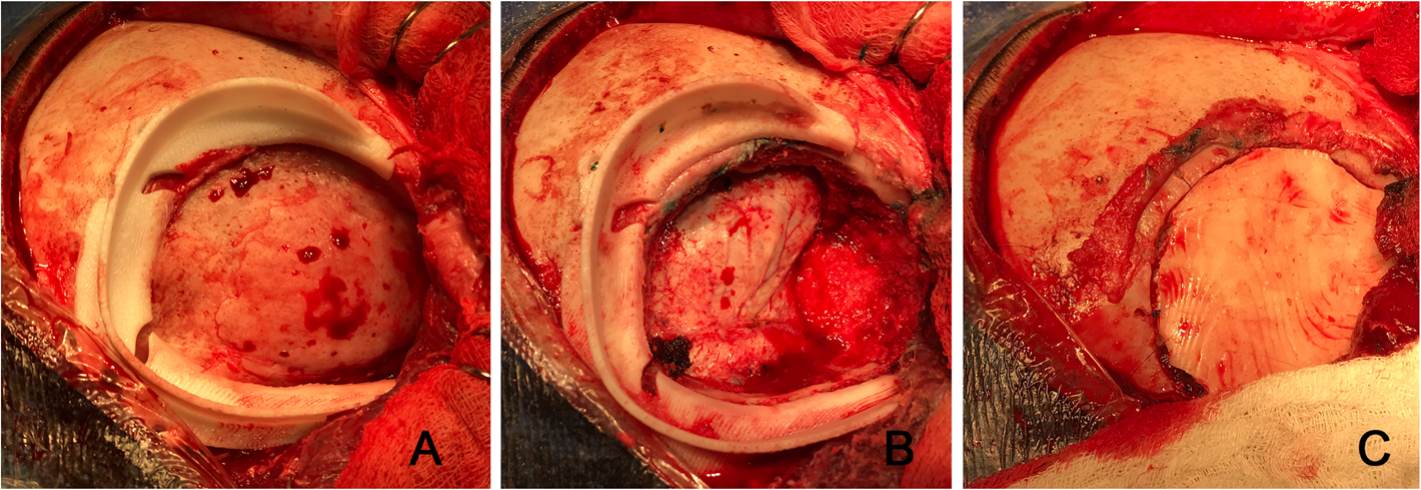
Illustrations of a single-step frame-guided resection: A. frame template placed delimiting the area of resection margins; B. frame template after bone removal; C. after cranioplasty

Upon being delivered to the hospital, a two-piece mould, craniectomy defect model, and test implant underwent sterilisation by steam autoclaving within 24 to 48 h prior to surgery. Patient care (anaesthesia induction, positioning) was performed as routine. Skin incision was created using a previous surgical scar in all patients. Craniectomy borders were exposed entirely (Fig. 6). The test implant was placed over the defect to check fitting quality, confirming no evidence of unevenness or gap between the bone and prosthesis (Fig. 7). After verification, the PMMA was prepared in a two-piece mould (Fig. 8). Once hardened after the thermo-reaction, the implant was compared with the test implant. Several random small holes were drilled in the implant for assimilation with the tissues (Fig. 9). Finally, it was placed over the defect and fixed in the skull with mini titanium plates (Fig. 10). For patients 9 and 11, the single-step frame-guided resection intraoperative details are shown in Fig. 11.

### Post-operative care

A high-resolution CT scan was performed to visualise the aesthetic result and symmetry. The patients were discharged 24 h after surgery.

### Patient’s perception of the final result of cranioplasty

To assess the cosmetic results, all patients were asked if they were satisfied with the result 2 months after surgery, responding objectively with YES or NO. One patient was unable to respond owing to her neurological condition, and her family members’ response was considered.

## Results

**Fig. 12:**
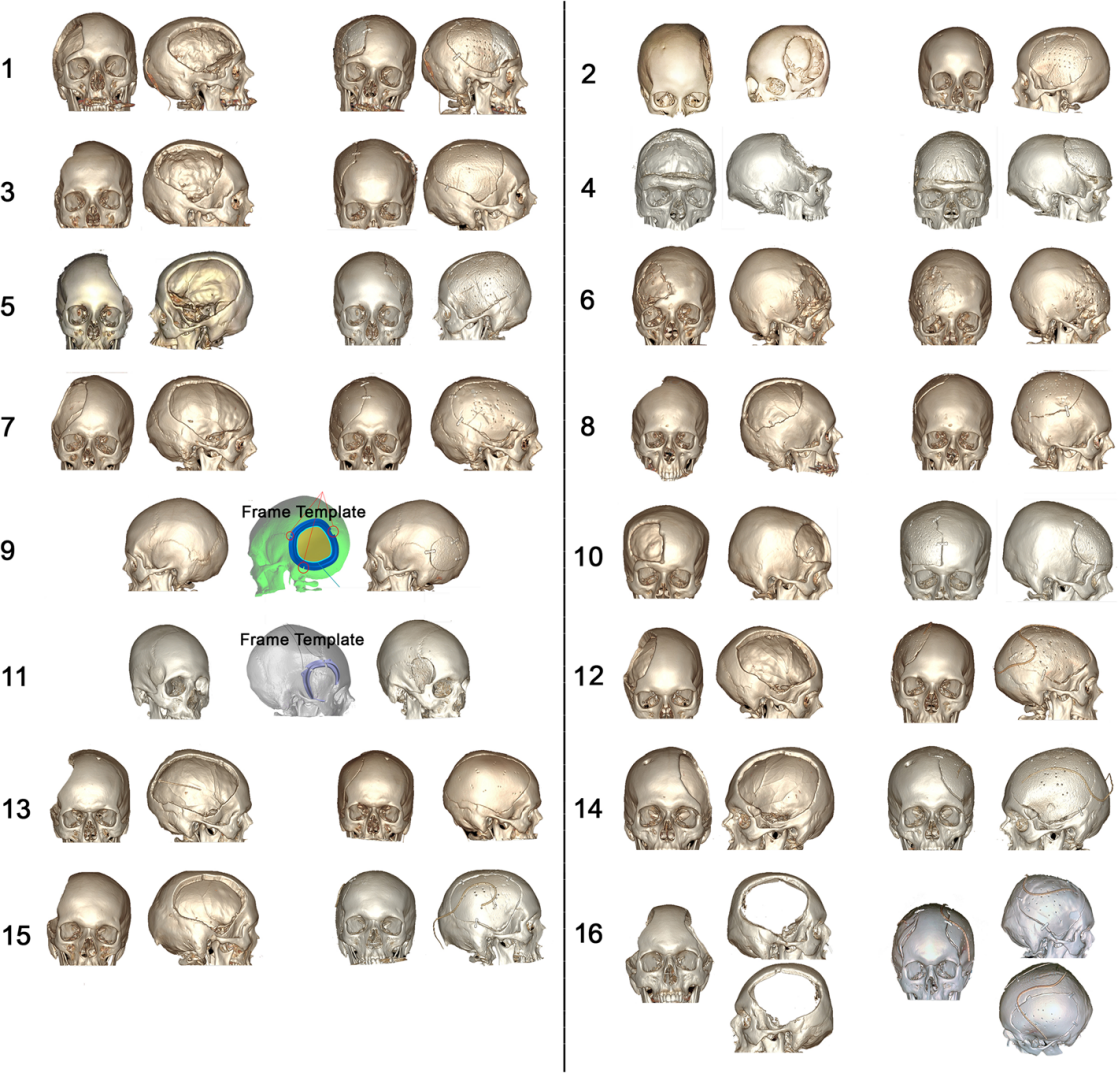
Pre-operative (coronal/sagittal) vs. post-operative (coronal/sagittal) of each patient. In patients 9 and 11, template frame was shown between pre and post-operative image

Table 1 shows each patient’s sex, age, cause of defect, defect location, size of the defect, total time for production, and implant thickness. A comparison between pre- and postoperative 3D CT scans of all patients is shown in Fig. 12. CAD/CAM treatment was performed in 5 to 6 days and 3D printing in approximately 20 h, plus 4–5 days for template cleaning, sterilisation, and delivery. No complications were observed during each planning. Costs per implant were approximately 6300 dollars (37,000 reais).

**Table 1:** Patients’ descriptions

Patient #	Sex	Age (years)	Cause of defect	Defect location	Size of the defect (cm)	Total time for production	Implant thickness
**1**	F	67	DC for brain infarction	Right frontotemporoparietal	10x13x3,5	11 days	0,2 cm
**2**	F	15	DC for intracranial hemorrhage	Left frontotemporoparietal	9,5 × 10,2 × 4,8	11 days	0,2 cm
**3**	M	35	DC for brain edema	Right frontotemporoparietal	10,25 × 12,3 × 3	10 days	0,2 cm
**4**	M	19	Osseous dysplasia and local infection	Bifrontal	12,7 × 12,3 × 6,2	11 days	2,8 cm
**5**	F	42	DC for brain edema	Left frontotemporoparietal	13,5 × 11,7 × 3,3	10 days	0,2 cm
**6**	F	42	DC for brain edema	Right frontal	9,3 × 8,7 × 2,2	9 days	0,2 cm
**7**	M	32	DC for trauma	Right frontotemporoparietal	11,5 × 14,6 × 2,9	11 days	0,2 cm
**8**	M	63	DC for brain tumor	Right parietal	8x12x3,2	9 days	0,2 cm
**9**	F	42	Intraosseous meningioma	Left retrosigmoid	6 × 5,9 × 1,2	10 days	0,4 cm
**10**	F	51	DC for brain infarction	Right frontal	7,5 × 9,2 × 2,4	9 days	0,2 cm
**11**	M	19	Fibrous dysplasia	Right sphenoidal	6 × 4,7 × 0,9	9 days	0,3 cm
**12**	F	23	DC for intracranial hemorrhage	Right frontotemporoparietal	14x11x2,1	10 days	0,2 cm
**13**	M	48	DC for intracranial hemorrhage	Right frontotemporoparietal	16x11x2,8	10 days	0,2 cm
**14**	M	57	DC for brain infarction	Left frontotemporoparietal	15x11x2,7	9 days	0,2 cm
**15**	M	58	DC for brain infarction	Right frontotemporoparietal	13x10x2,8	9 days	0,2 cm
**16**	M	18	DC for brain edema	Frontoparietal bilateral	14x10x2,3 and 13x10x2,3	9 days	0,2 cm

In the first patient, it was necessary to remake the implant a second time during the surgery. This occurred because the plate was deformed over removal from the mould before being completely hardened. Minor adjustments with drill to remove rough edges around the implant were performed in the first two patients due to a learning curve of the method. In subsequent cases, this was considered unnecessary for the aesthetic result since it did not interfere neither with the fitting nor the thickness of the implant. Post-operative infection, bleeding, or necessity for reoperation was not observed. All 16 patients and/or a relative answered YES regarding satisfaction with the implant.

### Patient’s follow-up

The shortest and longest postoperative follow-up were 3 and 26 months, respectively. There were no complications or late aesthetic deformities to date. Patients 1,2,5 and 7 have had atrophy of the temporal muscle evident in the immediate postoperative period. In those cases, the asymmetry on the operated side was evident, despite the good result of cranioplasty. In no case was it enough to change the positive assessment of patients and / or family members, or of the surgical team.

## Discussion

With the current paradigms of personalised medicine, various methods have come about to improve the aesthetic results of cranioplasties [7–9, 11–18]. This revolution in modern practice occurs due to the 3D printing of biomaterials and its disruptive applications in the twenty-first century medicine. Correction of cranial defects is a perfect example of this technological application: a closed, rigid, and immovable compartment with a defect that can be corrected by overlapping a simple prosthesis.

Although autogenous bone flaps are still the best option for defect correction, they are frequently unavailable for several reasons [2, 4, 5, 7, 19, 20]. Various studies have shown the advantages and disadvantages of every material used for cranioplasty [1, 7]. Infection rates may vary among patients receiving custom implants, and infection is still the most common complication in cranioplasty surgery with variable incidence rates. Regardless of the selected method, the timing of cranioplasty, patient’s performance, choice of the material, and surgical running time affect the risk of complications [2–5, 7, 21]. PMMA often exhibits low complication rates in cranioplasty [3, 9–11, 13, 22–24]. Although not described in the results, in our sample the shortest and longest surgical time were 42 and 65 min, respectively. Comparing with the previous surgeries in our institution without customized implants, there is an apparent reduction of 20 to 30 min in the operative time. Such information is an estimate for practical reference only. Infections or complications in the 16 patients from this study were not observed.

The inflated costs for a high-quality custom template [15, 25, 26] may be directly associated with bureaucracy, health systems limitations, and the lack of specific certified manufacturing processes in Brazil. Several studies have demonstrated the feasibility of producing low-cost custom implants, offering significant potential for cost savings and improving aesthetic results and patients’ quality of life [11, 14, 17, 27–30]. However, solutions that match the prominent level of medical technology available with optimised costs are still required.

Interdisciplinary collaboration between engineering and neurosurgery is an evident starting point. This concept, as previously described [14, 17, 18, 29, 30], favoured the creation of the mould. The use of 3D images facilitated the integration between medical staff and engineering. As observed in several articles [6, 9–18], the various CAD/CAM techniques offer safe and satisfactory aesthetic results regardless of the implanted biomaterial, provided that an appropriate scientific methodology is followed. In our experience, the ideal algorithm for mould production was observed when the surgeon adequately expressed his/her need to the engineering team via a medical phantom.

Following technological development, there are complex and rigorous regulatory issues specific to a particular country. Accessibility and regulatory compliance for 3D custom implants still lacks proper validation in Brazil. This makes the use of modern biomaterials temporarily unfeasible, which are still pending approval by the *Agência Nacional de Vigilância Sanitária*. This is a bureaucratic step that involves long-term efforts and needs to be fulfilled.

While the regulation of some biomaterials does not occur, the confection of 3D printed moulds for customised PMMA implants has been described as an alternative solution [23, 27, 29, 31]. In the manufacturing process, the cost of material to produce moulds is similar to that of the prosthesis. By automating the interdisciplinary design of implants during their manufacture under validated systems, the application of 3D printing could be routinely used in clinical practice while continuously overcoming the limitations [15, 16]. Product production, whether mould or implant, is achievable in less than 14 days. For the present article, up to 7 days from image acquisition to sterilisation has been fully possible.

The fight against bureaucracy and overpricing has become the next challenge. In the current Brazilian model, there is often an intermediary responsible for supplying products, adding a significant increase in the final value. In early 2017, three possible suppliers for custom cranioplasty templates were listed at our institution. None had legal regulations consistent with the use of biomaterials or appropriate specifications regarding implant production. The cost (implant only) to the patient or to the health insurance ranged from 14,000 dollars (70,000 reais) to 44,000 dollars (220,000 reais). Even considering Brazilian taxes, such prices are 2 to 7 times more expensive than expected in other countries [32–34] and often evolve into judicialization, harming all parties involved, specifically the patient. In the proposed method, an implant cost of less than 8000 dollars was achieved, without the need to differentiate costs by the dimensions of the templates. It included research, production, materials (polycarbonate templates and PMMA for the implant) and printing. In our institution, the hospital costs incurred for elective cranioplasty ranged from 3500 to 4000 dollars (18,000 to 20,000 reais). This implies an estimated final cost around 12,000 dollars (61,000 reais) for each patient or for the health insurance.

3D implants must undergo strict surveillance to ensure reliability and safety [35–37]. When printed by a company and sold to the hospital, they will be subject to regulation by health agencies. This validation process has an impact on the final cost of the product, especially when passed on to health insurers. Despite having an increasingly greater relevance and better cost-benefit ratio [38], the in-hospital production of 3D prints still requires more objective regulation, since it is treated on a case-by-case basis by regulatory agencies. This impacts the reliability and safety of 3D printing. In Brazil, this is especially challenging given the difficulty in implementing the quality of low-cost solutions.

Although it seems utopian, the integration and exchange of academic knowledge between health professionals, industry and government regulatory agencies can be an efficient solution to reduce bureaucracy in the customized implant manufacturing process, thus reducing costs, and benefiting patients.

We advocate that a transparent and high-quality solution under the scientific method can be cost-effective. If intermediary supplier bias is excluded, the entire system can benefit from reduced costs. Therefore, such technology could continue to evolve, focusing on welfare.

## Conclusion

Cranioplasty with high-technology customised 3D moulds for PMMA implants can achieve symmetric and aesthetic results, possibly with low complication rates. Systematisation of the entire manufacturing process leads to a fast and cost-effective process.

## Data Availability

The datasets during and/or analysed during the current study available from the corresponding author on reasonable request.
